# Formation of tubules and helical ribbons by ceramide phosphoethanolamine-containing membranes

**DOI:** 10.1038/s41598-019-42247-1

**Published:** 2019-04-09

**Authors:** Takehiko Inaba, Motohide Murate, Nario Tomishige, Yan-Fen Lee, Françoise Hullin-Matsuda, Brigitte Pollet, Nicolas Humbert, Yves Mély, Yasushi Sako, Peter Greimel, Toshihide Kobayashi

**Affiliations:** 1 Lipid Biology Laboratory, RIKEN, 2-1, Hirosawa, Wako, Saitama, 351-0198 Japan; 2 Cellular Informatics Laboratory, RIKEN, 2-1, Hirosawa, Wako, Saitama, 351-0198 Japan; 3 Laboratoire de Bioimagerie et Pathologies, UMR 7021 CNRS, Université de Strasbourg, Faculté de Pharmacie, 74 route du Rhin, 67401 Illkirch, France; 4 USM-RIKEN International Centre for Ageing Science, 11800 USM Penang, Malaysia; 5 School of Pharmaceutical Sciences, Universiti Sains Malaysia, 11800 Penang, Malaysia; 6 Université de Lyon, CarMeN laboratory, INSERM U1060, 69621 Villeurbanne, France; 7 Laboratory for Cell Function Dynamics, RIKEN Brain Science Institute, Wako, Saitama, 351-0198 Japan

## Abstract

Ceramide phosphoethanolamine (CPE), a major sphingolipid in invertebrates, is crucial for axonal ensheathment in *Drosophila*. Darkfield microscopy revealed that an equimolar mixture of bovine buttermilk CPE (milk CPE) and 1,2-dioleoyl-*sn*-glycero-3-phosphocholine (diC18:1 PC) tends to form tubules and helical ribbons, while pure milk CPE mainly exhibits amorphous aggregates and, at low frequency, straight needles. Negative staining electron microscopy indicated that helices and tubules were composed of multilayered 5–10 nm thick slab-like structures. Using different molecular species of PC and CPE, we demonstrated that the acyl chain length of CPE but not of PC is crucial for the formation of tubules and helices in equimolar mixtures. Incubation of the lipid suspensions at the respective phase transition temperature of CPE facilitated the formation of both tubules and helices, suggesting a dynamic lipid rearrangement during formation. Substituting diC18:1 PC with diC18:1 PE or diC18:1 PS failed to form tubules and helices. As hydrated galactosylceramide (GalCer), a major lipid in mammalian myelin, has been reported to spontaneously form tubules and helices, it is believed that the ensheathment of axons in mammals and *Drosophila* is based on similar physical processes with different lipids.

## Introduction

**Figure 1:**
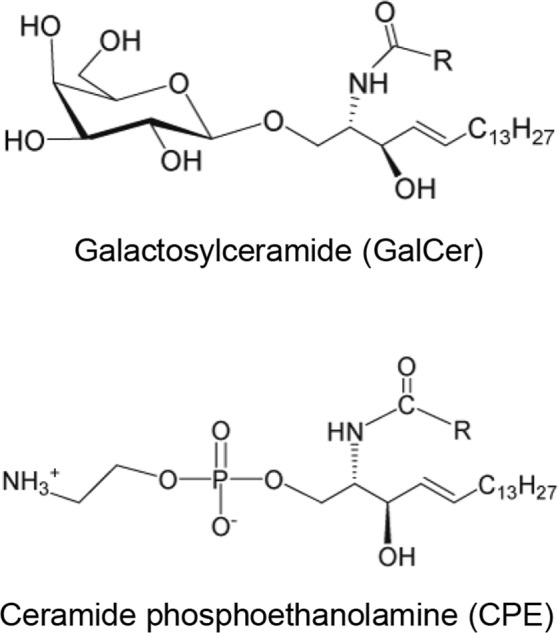
Structure of galactosylceramide (GalCer) and ceramide phosphoethanolamine (CPE).

The vertebrate nervous system is characterized by ensheathment of axons with myelin, a multilamellar membrane enriched in galactosylceramide (GalCer, Fig. 1). GalCer is one of the few natural lipids that forms helical ribbons in aqueous solution^1–4^. These helical ribbon structures have been postulated to be stabilized by intermolecular hydrogen bonds between GalCer molecules^3^. Although the detailed molecular mechanism is not fully understood, it has been speculated that planar lipid sheets roll up into cochleate cylinders to form myelin.

Unlike vertebrates, invertebrates such as *Drosophila* do not have myelin, but specialized glial cells that ensheath individual axons and fascicles of axons^5^. Recently, ceramide phosphoethanolamine (CPE) (Fig. 1) was identified as a key player in axonal wrapping by glia in *Drosophila*^6^. CPE enrichment in glial cells was demonstrated biochemically^6^ and histochemically using the specific CPE-binding protein, pleurotolysin A2 (PlyA2)^7^.

CPE is a phosphosphingolipid analog of sphingomyelin (SM) featuring a phosphoethanolamine instead of a phosphocholine headgroup^8^. CPE is the major sphingolipid in insects, such as *Drosophila*^9^, and in some protozoa parasites, such as *Trypanosoma brucei*^10^. In contrast, CPE represents only 0.002 to 0.02 mole % of total phospholipids in mammalian cells^11^ and tissues^12^.

In mammalian cells, sphingolipids such as SM, are thought to form specific “lipid raft” complexes with cholesterol (Chol)^13–15^. In contrast to SM, CPE does not form complexes with Chol^16,17^, possibly due to the less favorable CPE-Chol interaction compared to SM-Chol. This difference has been attributed to the smaller size of the CPE headgroup and its reduced efficiency to shield the sterol molecule from unfavorable water interaction^8,18^. On the other hand, the small size of the CPE headgroup allows stronger intermolecular hydrogen bonding between CPE molecules compared to SM, as highlighted by its elevated phase transition temperature^8,18^.

The morphology of CPE-containing membranes has not been studied in detail. Here, we report that CPE:phosphatidylcholine (PC) mixtures tend to form tubules and helical ribbons. The phase transition temperature of PC did not significantly affect the formation of helices whereas replacing PC by phosphatidylethanolamine (PE) or phosphatidylserine (PS) abolished the formation of tubules and helices. Varying the CPE *N*-acyl chain length in equimolar mixtures with 1,2-dioleoyl-*sn*-glycero-3-phosphocholine (diC18:1 PC) revealed CPE with very long *N*-acyl chains favored the formation of helices. In contrast, mixtures with medium and long *N*-acyl chain CPEs formed tubules. Based on our results, the formation of helical structures is related to strong intermolecular hydrogen bonds between CPE molecules and the hydrophobic mismatch between CPE and PC acyl chains.

## Results

**Figure 2:**
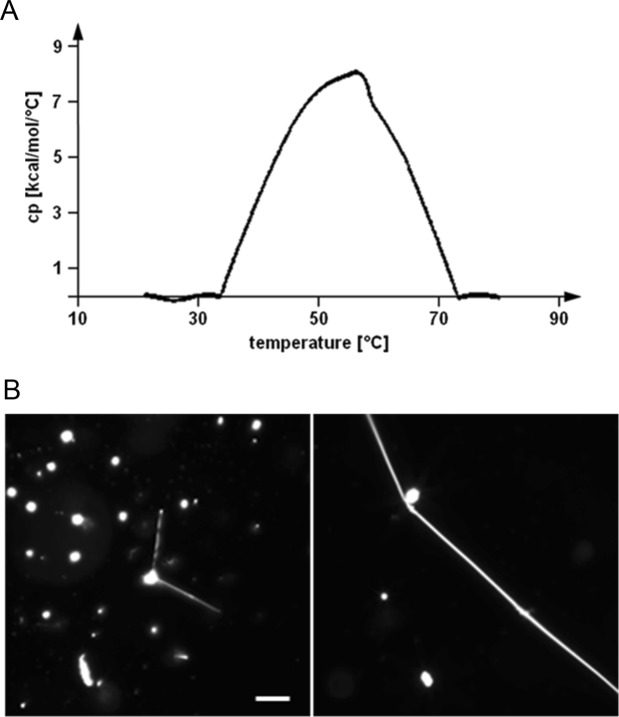
DSC heating thermograph and darkfield optical micrographs of milk CPE suspension. (**A**) Representative DSC heating thermograph of milk CPE (0.5 mM) dispersion in HEPES buffer. Scan rate, 10 degrees/h; scan range, 20–80 °C. (**B**) Milk CPE was hydrated at 60 °C as described in Materials and Methods. Darkfield images were acquired at room temperature (22 °C). Milk CPE exhibits amorphous lipid aggregates but needle and helical structures were also observed at low frequency. Bar, 50 μm.

We examined the morphology of CPE-containing membranes using commercially available CPE obtained from bovine buttermilk (Fig. 1), *N*-acyl-sphingosine-1-phosphoethanolamine (milk CPE) (Matreya, Pleasant Gap, PA). According to the manufacturer, the sphingoid base of milk CPE is composed of 18 carbons (d18:1, with ‘d’ referring to ‘di’ and indicating the presence of two hydroxy groups, followed by the carbon chain length and number of double bonds). The *N*-linked acyl chain length composition was determined by gas chromatography as follows (mol %): 16:0, 3.0%; 18:0, 3.9%; 20:0, 2.8%; 20:3, 2.2%; 22:0, 35.8%; 23:0, 29.7%; 24:0, 18.3%; 24:1, 3.1%; unidentified, 1.2%. Differential scanning calorimetry (DSC) showed a broad phase transition with a peak value at around 56 °C (Fig. 2A).

**Figure 3:**
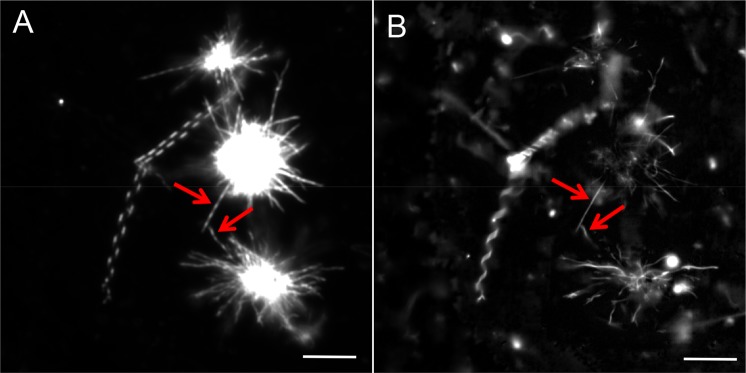
Darkfield (**A**) and fluorescence (**B**) micrographs of a dispersion of milk CPE:diC18:1 PC:DiI C18 (50:50:0.1) in water. Milk CPE (75 nanomoles in chloroform/methanol), diC18:1 PC (75 nanomoles in chloroform) and DiI C18 (0.15 nanomoles in chloroform) were aliquoted in a glass tube, solvents were removed under N_2_ flow and films were dried *in vacuo* as described in Materials and Methods. The lipid suspension was prepared at 60 °C as described in Materials and Methods. Darkfield and fluorescence images do not exactly overlap due to the sample movement between image acquisition. Red arrows indicate structures that are perceived as helical by darkfield and tubular by fluorescence microscopy. Bars, 10 μm.

**Figure 4:**
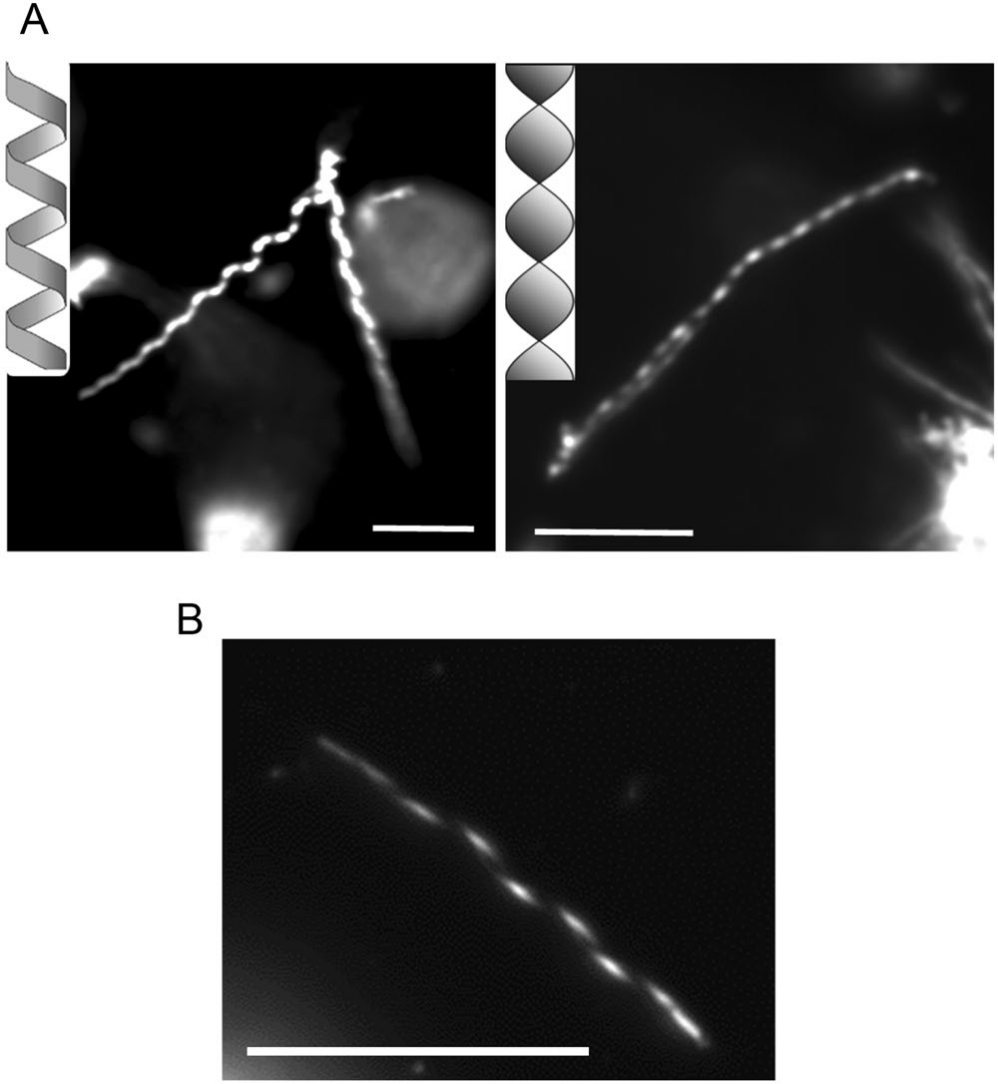
Dark field micrographs of an aqueous dispersion of milk CPE:diC18:1 PC (1:1) suspension. The lipid suspensions were prepared either with MilliQ water (**A**) or PBS (**B**) at 60 °C as described in Materials and Methods. Bars, 10 μm.

After hydration of milk CPE lipid films at 60 °C in MilliQ water, a large number of amorphous lipid aggregates were observed by darkfield microscopy. Additionally, needle and helix structures were also observed, albeit at much lower frequency (Fig. 2B). In contrast, lipid films composed of milk CPE and 1,2-dioleoyl-*sn*-glycero-3-phosphocholine (diC18:1 PC) were readily suspended in MilliQ water (Figs 3 and 4). In case of equimolar milk CPE:diC18:1 PC mixtures, helical ribbon assemblies were observed by darkfield microscopy (Fig. 3A). Helical ribbons were also observed by fluorescence microscopy upon addition of 0.1% of the fluorescent dye 1,1′-dioctadecyl-3,3,3′,3′-tetramethylindocarbocyanine perchlorate (DiI C18) (Fig. 3B). DiI C18 labeled the helical and tubular structures but not the bright lipid aggregates observed under darkfield microscopy (Fig. 3A). Previously we reported that DiI C18 partitioned into solid SM-rich domains in *N*-C16:0 SM/diC18:1 PC giant unilamellar vesicle (GUV)^19^. Therefore, our data in Fig. 3 suggest that helical structures are enriched with milk CPE whereas lipid aggregates are rich in diC18:1 PC. Moreover, DiI C18 labeled the whole helical structure, suggesting that both lipids were not phase-separated in the helix or phase separation occurred only below the resolution of optical microscopy. Several helical structures were only recognized under darkfield microscopy while corresponding fluorescence images revealed tubular structures (Fig. 3A,B, red arrows). Figure 4A shows that the diameters and pitches of the helices varied and that both helical ribbons (left) and twisted ribbons (right) were observed. Helical structures were observed both in MilliQ water and PBS (1.058 mM KH_2_PO_4_, 2.96 mM Na_2_PO_4_, 155 mM NaCl, pH 7.2) (Fig. 4B) and once formed, were stable at 4 °C for months.

**Figure 5:**
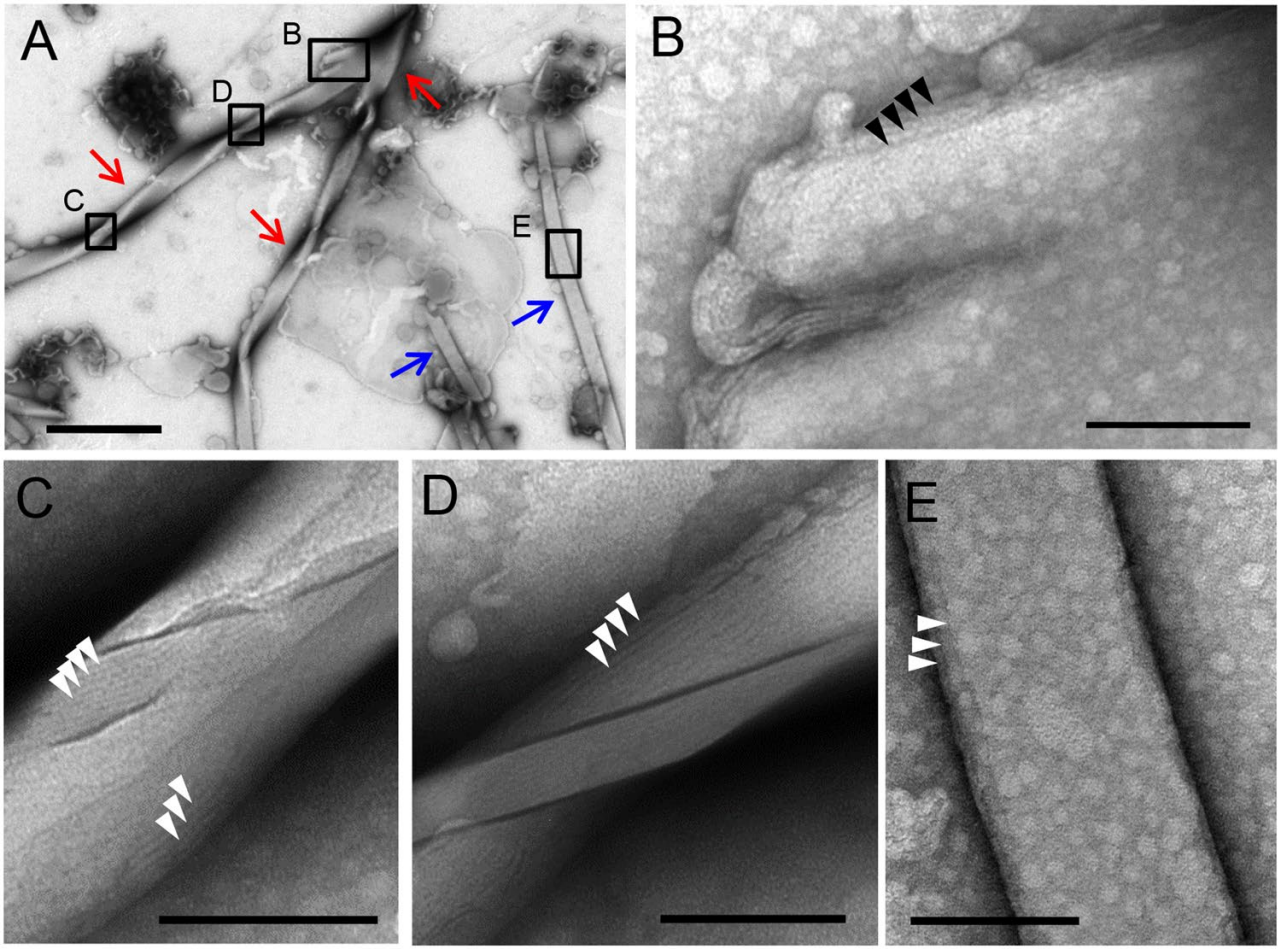
Negative staining electron micrographs of aqueous dispersion of milk CPE:diC18:1 PC (1:1). (**A**) Representative images of helical ribbons (red arrows) and tubules (blue arrows). (**B**) Magnified image of a branched area of a helix in (**A**). (**C**) and (**D**) Magnified images of helical pitches in (**A**). Stacks of lipid layers are observed in ribbons and tubules (arrowheads). (**E**) Magnified image of a tubule in (**A**). Bars, (**A**) 1 μm; (**B**–**E**) 100 nm.

Negative staining electron micrographs of hydrated milk CPE:diC18:1 PC (1:1) suspensions revealed both helices (red arrows in Fig. 5A) and tubules (blue arrows in Fig. 5A) with 100–500 nm width in addition to small aggregates. Figure 5A shows two helices originating from a single bigger helix. The close-up view revealed that the helices were composed of 5–10 nm thick slab-like multilayers (Fig. 5B–D,  arrowheads indicate each layer). Several layered structures were also observed in tubules (Fig. 5E, arrow heads).

**Figure 6:**
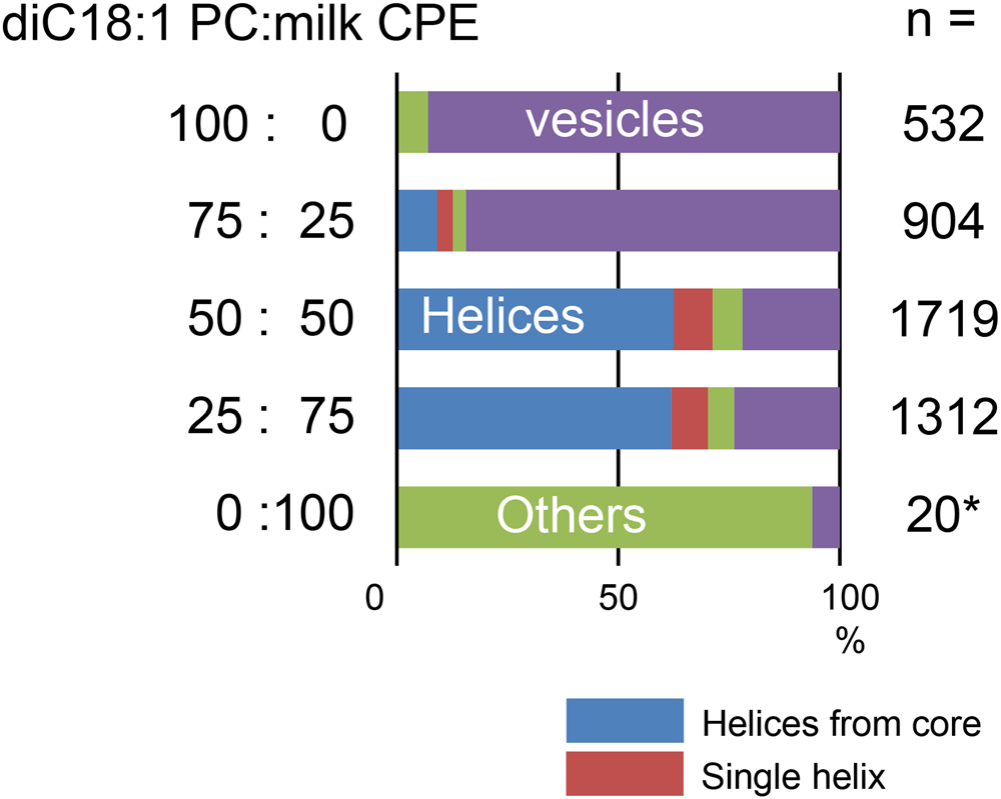
Morphology of membranes made of milk CPE:diC18:1 PC at different ratios. Red color describes the occurrence of single helices. Others correspond to amorphous aggregates. *Low sample number was examined due to difficulties during lipid hydration.

**Figure 7:**
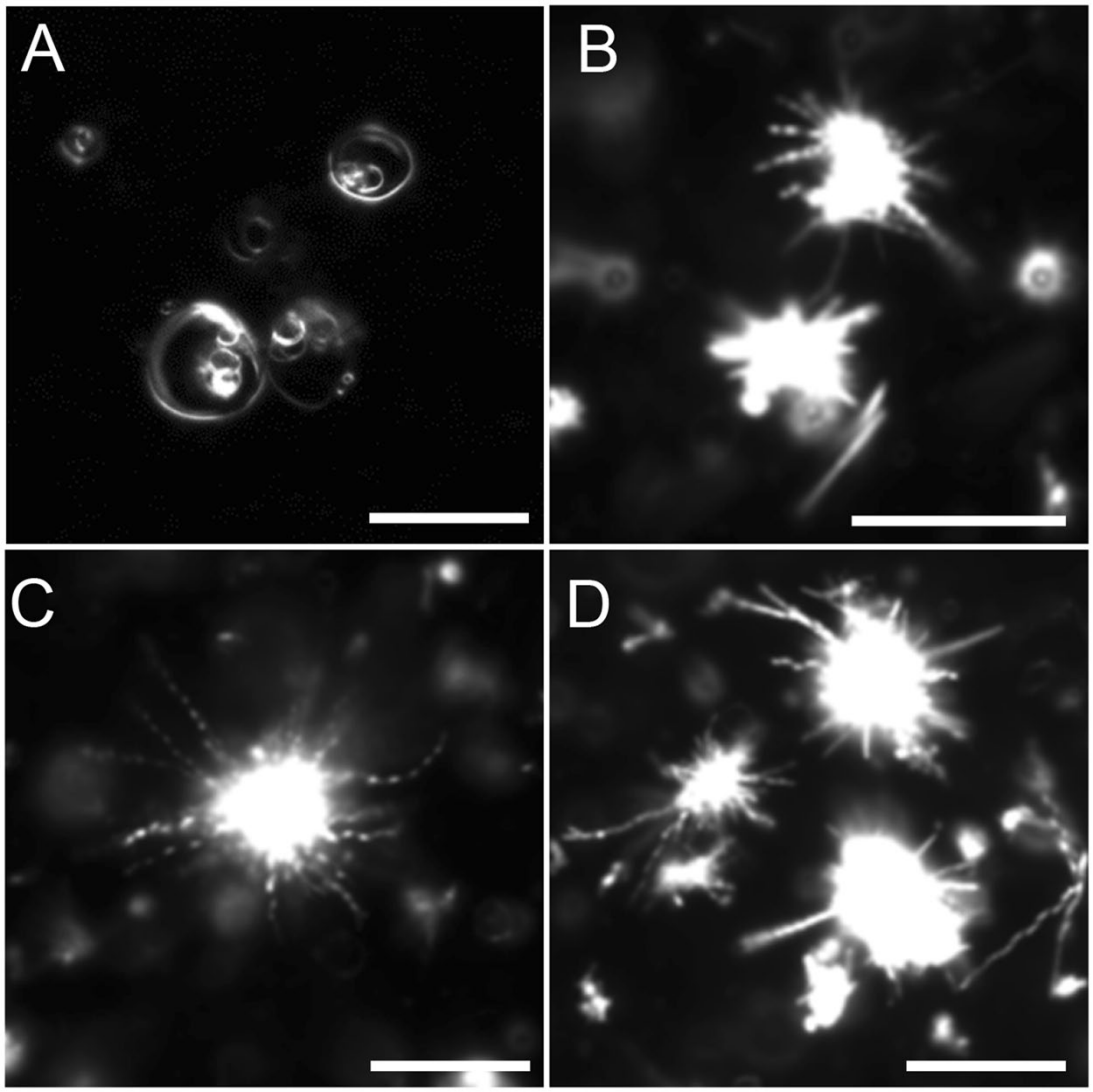
Darkfield optical micrographs of aqueous dispersion of (**A**) diC18:1 PC, (**B**) milk CPE:diC18:1 PC (1:1), (**C**) milk CPE:C16:0, C18:1 PC (1:1) and (**D**) milk CPE:diC16:0 PC (1:1) suspensions. The lipids were suspended in MilliQ water at 60 °C as described in Materials and Methods. Bars, 20 μm.

**Figure 8:**
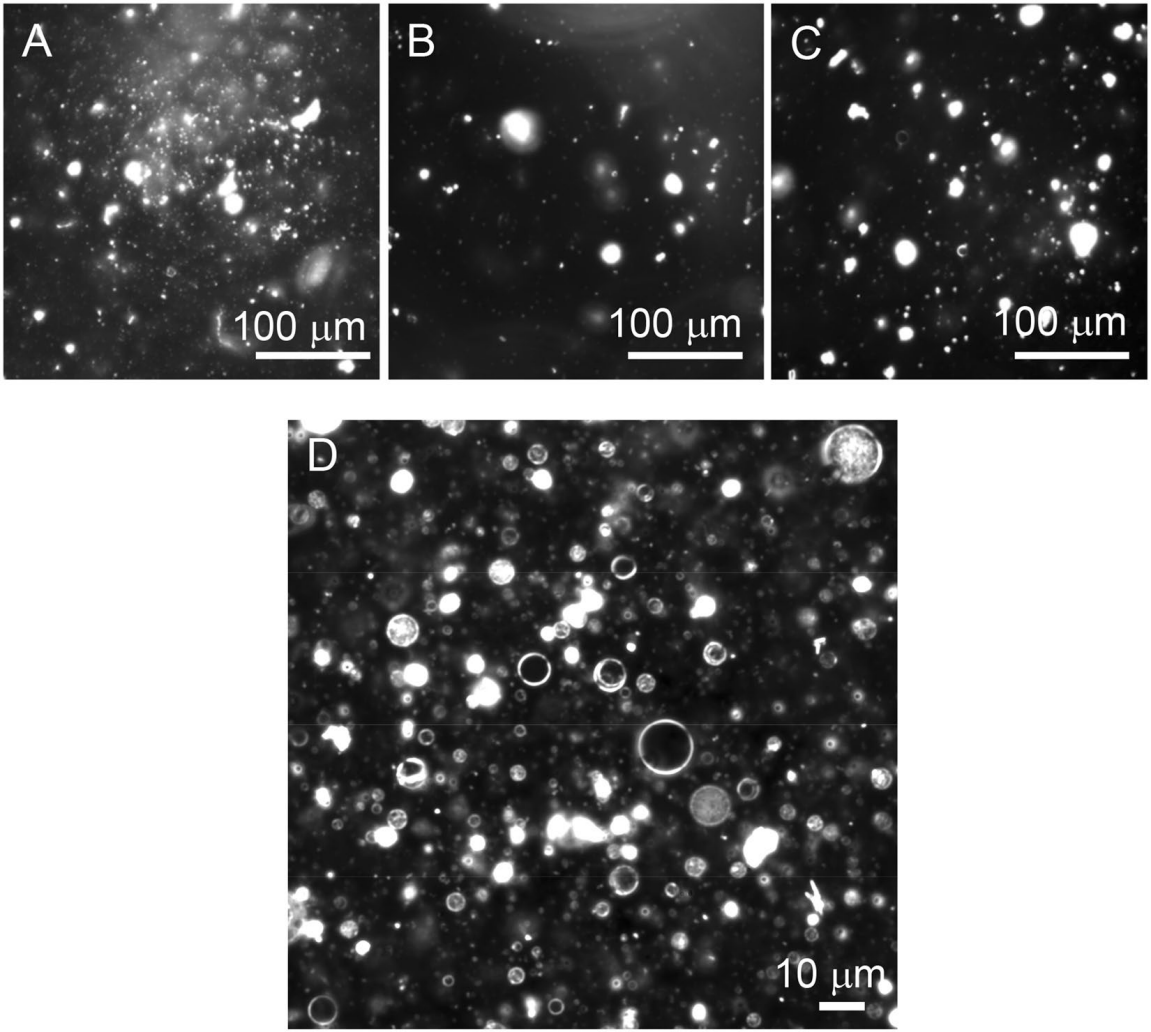
Darkfield optical micrographs of aqueous dispersion of (**A**) milk CPE:diC18:1 PE (1:1), (**B**) milk CPE:diC16:0 PE (1:1), (**C**) milk CPE:cholesterol (1:1) and (**D**) milk CPE:diC18:1 PS (1:1) suspensions. The lipid suspensions were prepared in MilliQ water at 60 °C as described in Materials and Methods.

Figure 6 summarizes the observed morphology of milk CPE:diC18:1 PC lipid suspensions at different ratios. While pure CPE exhibited mainly amorphous aggregates (Figs 2 and 6), its mixtures with diC18:1 PC were associated with the formation of helical structures, as observed by darkfield microscopy (Fig. 6). The morphology of the lipid suspensions at 50% and 75% milk CPE were very similar, showing a large majority of helical structures and a significant percentage of single helix. A strong reduction of the helical fraction was observed upon decreasing milk CPE content from 50 to 25%. Pure diC18:1 PC mainly formed vesicles in milliQ water (Figs 6 and 7A). Similar to equimolar milk CPE: diC18:1 PC mixtures (Fig. 7B), milk CPE mixed with 1-palmitoyl-2-oleoyl-*sn*-glycero-3-phosphocholine (C16:0, C18:1 PC) (Fig. 7C) or 1,2-dipalmitoyl-*sn*-glycero-3-phosphocholine (diC16:0 PC) (Fig. 7D) formed helical ribbons. In contrast, equimolar milk CPE mixtures with 1,2-dioleoyl-*sn*-glycero-3-phosphoethanolamine (diC18:1 PE) (Fig. 8A), 1,2-dipalmitoyl-*sn*-glycero-3-phosphoethanolamine (diC16:0 PE) (Fig. 8B) or cholesterol (Fig. 8C) did not form helical ribbons but rather amorphous aggregates. Moreover, equimolar milk CPE:1,2-dioleoyl-*sn*-glycero-3-phosphoserine (diC18:1 PS) suspensions gave rise to vesiclular structures (Fig. 8D).

**Figure 9:**
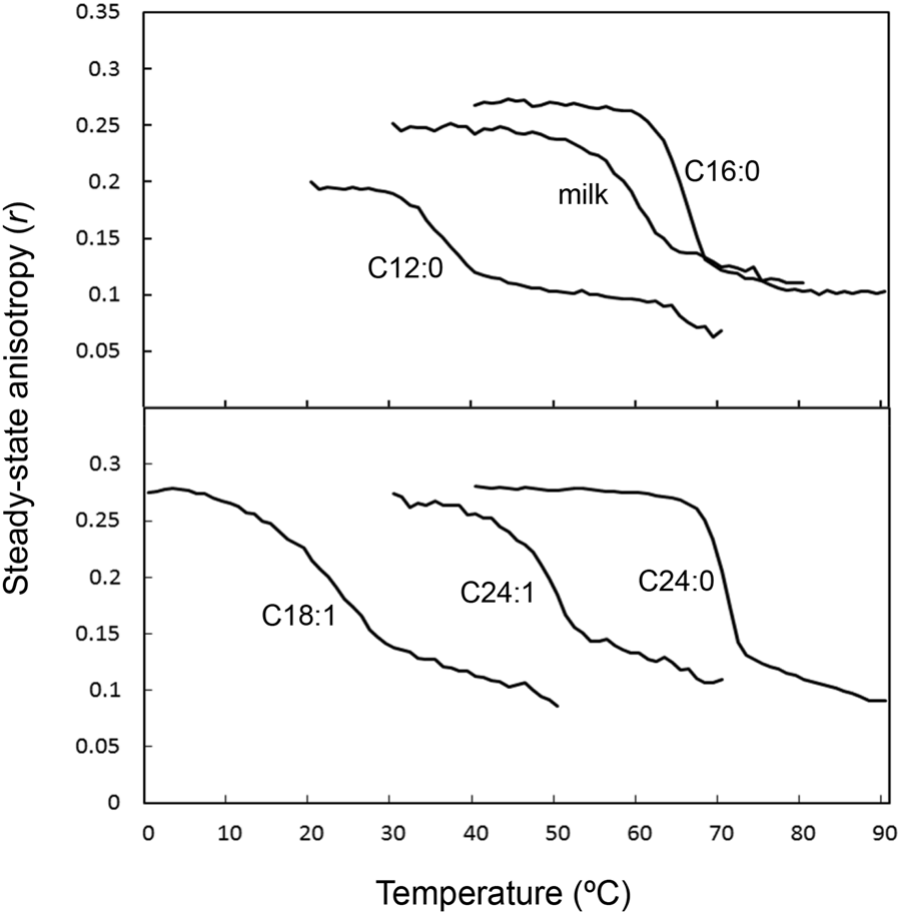
Steady-state anisotropy (*r*) of DPH in various CPE-based suspensions. Lipid suspensions were prepared from pure CPEs as described in Materials and Methods. The anisotropy of DPH is drawn as a function of the temperature. C12:0, d17:1/*N*-12:0 CPE; milk, milk CPE; C16:0, d18:1/*N*-16:0 CPE; C18:1, *N*-18:1 CPE; C24:1, d18:1/*N*-24:1^Δ15 (c)^ CPE; C24:0, d18:1/*N*-24:0 CPE.

Next we examined the effect of the CPE *N*-acyl chain length on the morphology of CPE:diC18:1 PC suspensions. The following CPE species were studied: *N*-lauroyl sphingosylphosphorylethanolamine (d17:1/*N*-12:0 CPE) (Avanti Polar Lipids), *N*-palmitoyl sphingosylphosphorylethanolamine (d18:1/*N*-16:0 CPE) and *N*-oleoyl sphingosylphosphorylethanolamine (*N*-18:1 CPE) (enzymatically synthesized from d18:1/*N*-16:0 SM and *N*-18:1 SM^16^), and *N*-lignoceroyl sphingosylphosphorylethanolamine (d18:1/*N*-24:0 CPE) and *N*-nervonoyl sphingosylphosphorylethanolamine (d18:1/*N*-24:1^Δ15 (c)^ CPE) (custom synthesized by Avanti Polar Lipids). As the physical properties of above listed CPEs have not been reported previously, except for d18:1/*N*-16:0 CPE^16^, we measured the anisotropy of 1,6-diphenyl-1,3,5-hexatriene (DPH) as a function of temperature in bilayers of each CPE species in MilliQ water (Fig. 9). DPH orients itself along the acyl chains of the phospholipids in the bilayer and is sensitive to the packing properties in the hydrophobic core of the bilayer membrane^20^. CPEs with saturated *N*-acyl residues (d18:1/*N*-16:0 CPE and d18:1/*N*-24:0 CPE) showed a steep decrease in the anisotropy of DPH as the temperature was increased. In contrast, CPEs with unsaturated *N*-acyl residures (*N*-18:1 CPE and d18:1/*N*-24:1^Δ15 (c)^ CPE), short *N*-acyl chain (d17:1/*N*-12:0 CPE) or with mixed *N*-acyl chain length (milk CPE) showed a broad gel to liquid crystalline transition (Fig. 9). The mid temperature of the transition for *N*-18:1 CPE, d17:1/*N*-12:0 CPE, d18:1/*N*-24:1^Δ15 (c)^ CPE, milk CPE, d18:1/*N*-16:0 CPE and d18:1/*N*-24:0 CPE were 24 °C, 38 °C, 50 °C, 59 °C, 63 °C and 72 °C, respectively. The phase transition temperature of d18:1/*N*-16:0 CPE (63 °C) is in good agreement with the previously reported data (64 °C)^16^ and the transition temperature of milk CPE (59 °C) was close to the phase transition temperature in HEPES dispersions measured by DSC (56 °C, Fig. 2).

**Figure 10:**
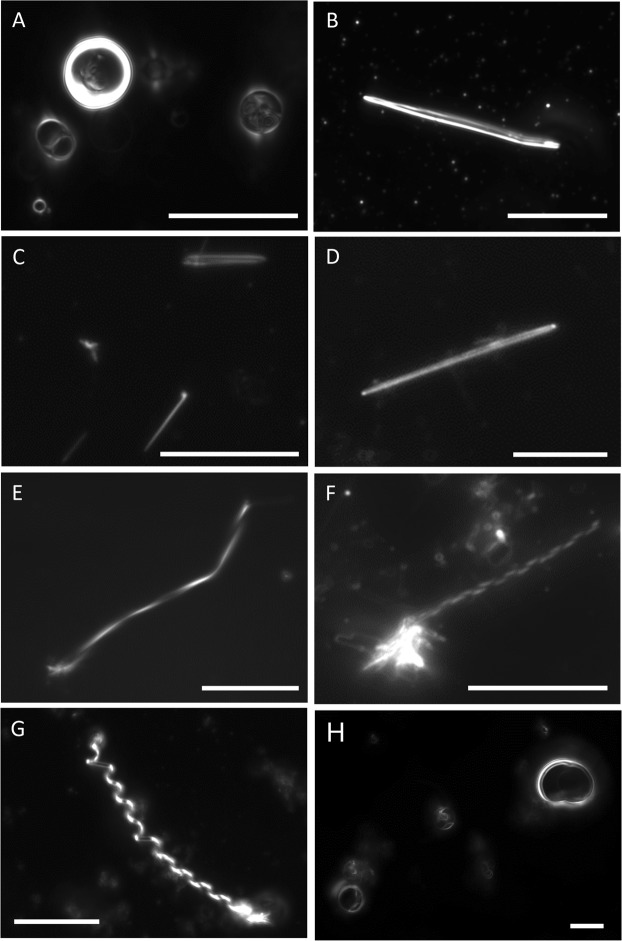
Darkfield optical micrographs of aqueous dispersions of various CPE:diC18:1 PC (1:1) suspensions. (**A**) diC18:1 PC, (**B**,**H**) d17:1/*N*-12:0 CPE:diC18:1 PC, (**C**) d18:1/*N*-16:0 CPE:diC18:1 PC, (**D**) *N*-18:1 CPE:diC18:1 PC, (**E**) d18:1/*N*-24:0 CPE:diC18:1 PC, (**F**) d18:1/*N*-24:1^Δ15 (c)^ CPE::diC18:1 PC, (**G**) milk CPE:diC18:1 PC. Lipid suspensions were prepared in MilliQ water at (**A**,**G**,**H**) 60 °C, (**B**) 32 °C, (**C**) 61 °C, (**D**) room temperature (22 °C), (**E**) 67 °C and 41 °C (**F**) as described in Materials and Methods. Bars, 10 μm.

Subsequently, the morphology of equimolar suspensions of each CPE species with diC18:1 PC  was examined (Fig. 10). Pure diC18:1 PC suspensions produced vesicles (Figs 7A and 10A), while equimolar mixtures with d17:1/*N*-12:0 CPE, d18:1/*N*-16:0 CPE or *N*-18:1 CPE formed tubules (Fig. 10B–D). In case of binary mixtures containing longer chain CPE, either d18:1/*N*-24:0 CPE or d18:1/*N*-24:1^Δ15 (c)^ CPE, helical structures (Fig. 10E,F) were observed similar to milk CPE:diC18:1 PC (1:1) suspensions (Fig. 10G). Importantly, the incubation temperature was critical for the formation of helical structures. Incubation of d18:1/*N*-24:1^Δ15 (c)^ CPE:diC18:1 PC (1:1) mixtures at the lower end of their transition temperature range, at 41 °C, facilitated helix formation. In contrast, incubation of the same lipid suspension at 60 °C yielded amorphous lipid aggregates (not shown). Equimolar mixtures of diC18:1 PC with d18:1/*N*-24:0 CPE or milk CPE formed helical structures when incubated around phase transition temperature, while mixtures containing d17:1/*N*-12:0 CPE, d18:1/*N*-16:0 CPE or *N*-18:1 CPE formed tubules. Incubated at 60 °C of the equimolar suspension of d17:1/*N*-12:0 CPE:diC18:1 PC exhibited vesiclular structures (Fig. 10H).

**Figure 11:**
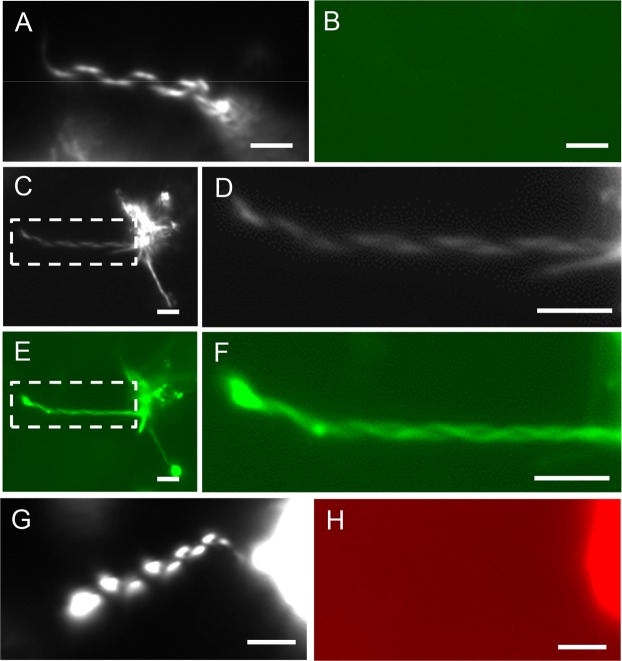
Binding of PlyA2 to CPE:diC18:1 PC (1:1) suspensions. (**A**,**B**) PlyA2-EGFP was added to pre-formed milk CPE:diC18:1 PC (1:1) helices at room temperature. Darkfield (**A**) and fluorescence (**B**) images were acquired after 30 min incubation. (**C**–**F**) PlyA2-EGFP was added to d18:1/*N*-24:1^Δ15 (c)^ CPE:diC18:1 PC during hydration of the lipid film at 41 °C for 3 h, followed by darkfield (**C**,**D**) and fluorescent (**E**,**F**) microscope analysis. Dotted squared area in (**C**) and (**E**) were enlarged in (**D**) and (**F**), respectively. (**G**,**H**) mCherry-NT-Lys was added to d18:1/*N*-24:1^Δ15 (c)^ CPE:diC18:1 PC as described above and darkfield (**G**) and fluorescent (**H**) images were acquired. Bars, 1 μm.

Previously we showed that the mushroom-derived protein, pleurotolysin A2 (PlyA2) binds to CPE but not PC^7^. Consequently, we used PlyA2-EGFP to examine the localization of CPE in helical structure. Addition of PlyA2-EGFP to already suspended milk CPE:diC18:1 PC (1:1) mixtures did not result in visible membrane labeling (Fig. 11A,B). In contrast, PlyA2-EGFP addition to d18:1/*N*-24:1^Δ15 (c)^ CPE:diC18:1 PC mixtures during lipid film hydration at 41 °C resulted in labeling of both helices and lipid aggregates (Fig. 11C–F). To probe whether PlyA2-EGFP labeling occurred due to non-specific inclusion of the fluorescent protein in the helices, we treated d18:1/*N*-24:1^Δ15 (c)^ CPE:diC18:1 PC mixtures with mCherry-non toxic-lysenin (mCherry-NT-Lys) during hydration. mCherry-NT-Lys specifically binds SM but not CPE or PC^7,21^. mCherry-NT-Lys weakly labeled lipid aggregates but not helices (Fig. 11G,H), excluding the possibility that PlyA2-EGFP labeling of helices was nonspecific.

## Discussion

CPE is enriched in *Drosophila* glial cells^6,7^ and is required for the development of cortex glia^22^. However, little is known about the physical properties of CPE. The present study shows that hydrated CPE:PC mixtures form vesicular, tubular and helical structures, depending on the *N*-linked acyl chain length and incubation conditions. The gel to liquid crystalline phase transition temperature of d18:1/*N*-16:0 CPE (64 °C) has been reported to be 23 degrees higher compared to d18:1/*N*-16:0 SM (41 °C)^16^ and is consistent with our results (63 °C). Similarly, the phase transition temperature of d18:1/*N*-24:0 CPE (72 °C) and d18:1/*N*-24:1^Δ15 (c)^ CPE (50 °C) is more than 20 degrees higher compared to their SM analogues *N*-24:0 SM (47.5 °C)^23^ and *N*-24:1^Δ15 (c)^ SM (22.3 °C)^3^. Similar differences in the physical properties of choline-containing lipids compared to ethanolamine-containing lipids were observed in glycerolipids, as the phase transition temperature of saturated PE is 20–30 degrees above the corresponding PC^24^. These differences are attributed to both the stronger headgroup interaction and the tighter packing owing to the small size of the headgroup and the intermolecular hydrogen bonding of PE molecules. By analogy with PE, the small headgroup size of CPE has been proposed to play a critical role to facilitate the strong CPE-CPE interaction^16^. This is in line with our recent report of stronger amide bond interaction between CPE molecules compared to SM molecules^17^.

Milk CPE exhibits a main phase transition temperature of 56–59 °C and the primary *N*-acyl lengths are 22:0 (35.8%), 23:0 (29.7%) and 24:0 (18.3%). Even above transition temperature, pure milk CPE films poorly detached from the tube walls and the resulting suspension exhibited amorphous aggregates. Needle and helical structures were observed at low frequency. In contrast, equimolar milk CPE: diC18:1 PC mixture was readily suspended in MilliQ water or PBS and exhibited helical structures. At the observing temperature 21–22 °C, milk CPE is in gel phase while diC18:1 PC (phase transition at −17.3 °C^25^) is in liquid crystalline phase. Consequently, phase separation of the two lipid phases would be expected. The fluorescent dye DiI C18 labeled helical and tubule structures, but not lipid aggregates formed in equimolar lipid suspensions. This suggests that helical structures are enriched with solid milk CPE whereas aggregates are diC18:1 PC-rich. Systematic variation of the milk CPE:diC18:1 PC ratio revealed that helical structures were dominant in mixtures with high CPE content, above 50%, consistent with the notion that helices are mainly composed of CPE. The homogeneously labeled helical structures by DiI C18 suggest that helical structures are not phase separated. However, the presence of phase separated domains below the resolution limit of optical microscopy in the helical structures cannot be excluded.

Similar to milk CPE: diC18:1 PC (1:1), helical structures were also observed in equimolar milk CPE/C16:0, C18:1 PC (phase transition at −4 °C^25^) and milk CPE/diC16:0 PC (phase transition at 41.4 °C^25^) suspensions. Interestingly, at the working temperature (21–22 °C), diC18:1 PC and C16:0, C18:1 PC are in liquid crystalline phase while diC16:0 PC is in gel phase. These results indicate that the phase properties of PC do not significantly affect the formation of helical structure in equimolar milk CPE:PC suspension.

**Figure 12:**
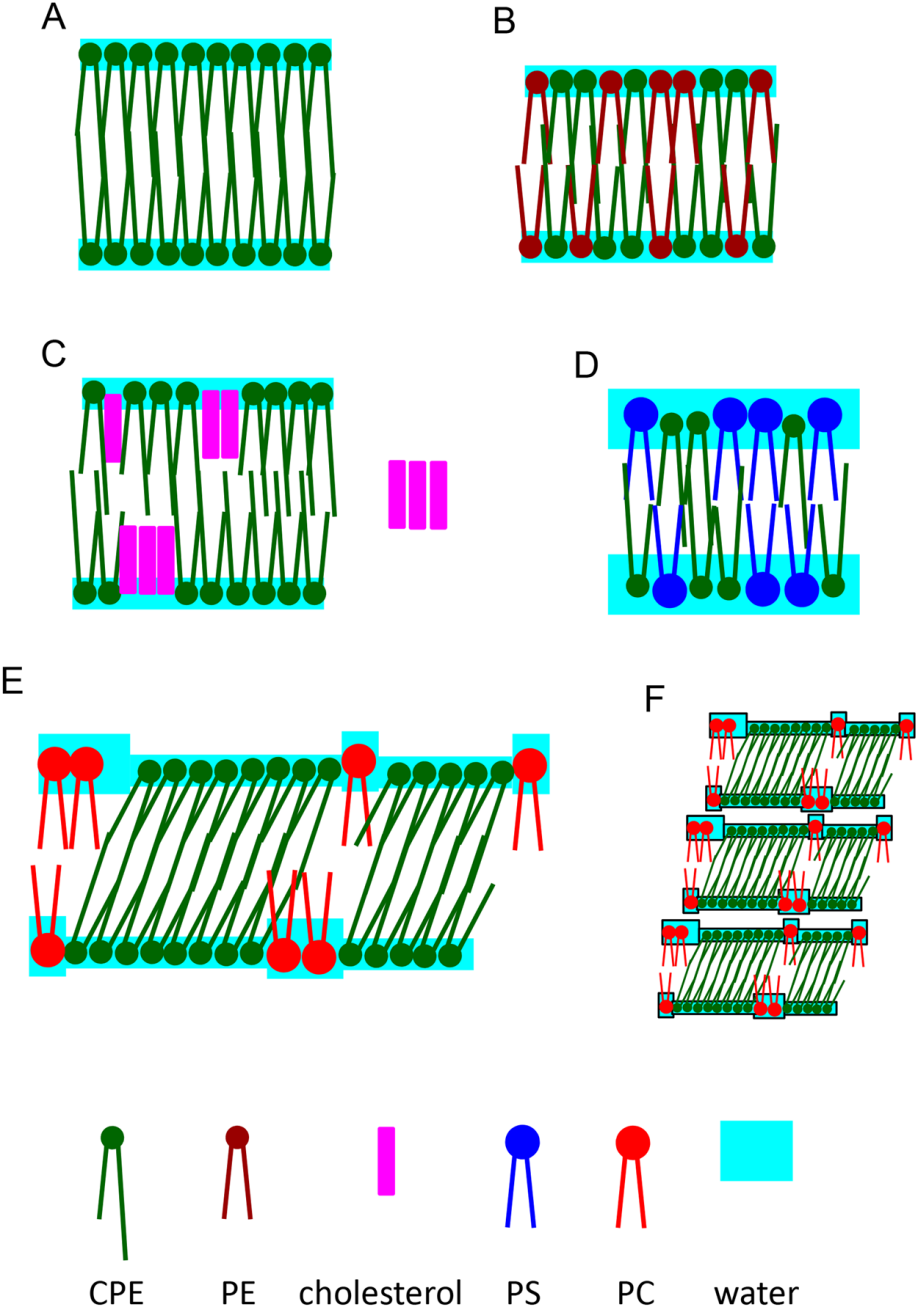
Schematic view of the distribution of CPE with very long *N*-acyl chains in different membranes. When lipid suspensions were prepared, PE, PS and PC were above phase transition temperatures whereas CPE was at the phase transition temperature. At observation temperature, PC and PE were either in solid or liquid crystalline phases, whereas CPE was in solid phase. The lipid phase of PC and PE did not affect the morphology of the membranes. (**A**) In CPE suspensions, the hydrogen bonds between the small headgroups and van der Waals interaction between the hydrophobic tails form tightly packed membranes that prevent hydration. The majority of CPE membranes show amorphous aggregates. (**B**) PE and CPE interact via hydrogen bonds of their headgoups. The membrane is tightly packed and forms aggregates. (**C**) Due to the low solubility of cholesterol in CPE, cholesterol is segregated from CPE in the membrane. Moreover, cholesterol can be excluded from the membrane and precipitate (in the right part). (**D**) Similar to PE, PS can form hydrogen bonds with CPE. However, the larger headgroup of PS allows the hydration of the membrane and thus, the formation of vesicles. (**E**) Unlike PE and PS, PC does not mix well with CPE. Rather, the two lipids are phase separated. In addition to large scale phase separation (aggregates enriched with PC and tubules and helices enriched with CPE), a small amount of PC exists in the helices and is phase separated from CPE. The PC molecules in the helices likely help to hydrate the membrane. Due to the hydrophobic mismatch of PC and CPE, the CPE domains are thought to tilt. CPE domains are stabilized by interdigitated interaction. (**F**) Thin layer of water phase in PC:CPE likely facilitates the piling up of membranes.

Unlike milkCPE:PC (1:1) suspensions, equimolar suspensions of milk CPE:diC18:1 PE, milk CPE:diC16:0 PE and milk CPE:cholesterol exhibited amorphous aggregates. It is speculated that the small headgroups of both CPE and PE favor tight interaction, impeding hydration of these mixtures (Fig. 12A,B). In case of CPE:cholesterol, the hydrated membranes likely coexist with cholesterol precipitates due to the low solubility of cholesterol in CPE^16,17^ (Fig. 12C). While CPE and PS can engage in intermolecular hydrogen bonding, the comparatively larger headgroup of PS likely facilitates hydration of the membrane. Consequently, it can be speculated that in the presence of PS the formation of CPE-rich lipid domains is prevented, precluding the formation of helical structures (Fig. 12D).

Equimolar suspensions of diC18:1 PC with d17:1/*N*-12:0 CPE (phase transition at 38 °C), d18:1/*N*-16:0 CPE (63 °C) and *N*-18:1 CPE (24 °C) formed tubules, while suspensions with d18:1/*N*-24:0 CPE (72 °C) and d18:1/*N*-24:1^Δ15 (c)^ CPE (50 °C) exhibited helical structures. No clear correlation between the phase transition temperature of the respective CPE and the formation of helical structures in equimolar CPE: diC18:1 PC suspension can be discerned. However, a correlation between *N*-acyl chain length of CPE and the preferred morphology of equimolar CPE: diC18:1 PC suspensions can be noticed, as medium and long *N*-acyl chain CPEs form tubules whereas very long chain CPEs form helices. It is noted that the main *N*-acyl chain lengths of milk CPE are 22:0, 23:0 and 24:0, constituting an enrichment with very long chain fatty acids. In *Drosophila*, the sphingolipid bases have shorter carbon chain length (d14:1 and d16:1) compared to mammals (d18:1)^26^. However, the most abundant CPE species features very long *N*-acyl chain (d14:1/*N*-20:0 CPE and d14:1/*N*-22:0 CPE)^22,26^.

Previously, aqueous suspensions of GalCer or GalCer:PC have been shown to form tubules and helices^1,3,27^. The morphology of these lipid suspensions was found to depend on the chain length of the *N*-acylated fatty acyl redisues. *N*-24:0 GalCer prefers to form large, variable-length ribbon-like structures, whereas *N*-16:0 GalCer and *N*-24:1 GalCer form cylindrical structures^1,3,27^. Similar to CPE, GalCer shows higher gel to liquid crystalline phase transition temperature compared to corresponding SM. The major phase transition temperatures are 59.3 °C and 84.1 °C for *N*-24:1 GalCer and *N*-24:0 GalCer, respectively^3^. It has been proposed that intermolecular hydrogen bonds play an important role in tubules and helices formation^3^. However, there are several differences between CPE and GalCer. First, GalCer alone forms helices and tubules whereas CPE forms these structures only when mixed with PC. Second, *N*-24:1 GalCer forms tubules whereas *N*-24:0 GalCer forms helices. In contrast, both d18:1/*N*-24:0 CPE:diC18:1 PC (1:1) and d18:1/*N*-24:1^Δ15 (c)^ CPE:diC18:1 PC (1:1) display helical structures. These results suggest that in addition to the strong hydrogen bonding between the lipid headgroups, other headgroup specific features play an additional role in the formation of tubules and helices.

Helices and tubules formed efficiently upon hydration at the phase transition temperature of the respective CPE. This suggests that dynamic lipid re-organization is required to form helical structures, as the transbilayer lipid movement is accelerated at the phase transition temperature^28^. We speculate that the hydrophobic mismatch between very long chain CPE and diC18:1 PC induces tilting of CPE rich membrane domains (Fig. 12E). Restraining the chiral CPE molecules to a bilayer phase while maintaining a tilt with respect to the local layer normal together with the favored twist of neighbor to neighbor interaction, guides the whole assembly to twist into a helical ribbon^3,29^. Within the plane of the monolayer, the strong linear lipid association necessary to stabilize the formed tubular and helical structures are likely supported by hydrogen bonding^3,29^. Interdigitated interaction between the very long chain CPEs in PC membrane may also stabilize the tilted CPE domains (Fig. 12E). Moreover, the thin water layer on CPE may facilitate the piling up of bilayer as observed by electron microscopy (Figs 5 and 12F).

The EGFP-tagged CPE-binding protein PlyA2 was unable to bind to preformed helical structures composed of milk CPE:diC18:1 PC. Since tight lipid packing was reported to prevent binding of lipid-binding peptides^30^, we speculate that CPE is tightly packed in helical structures. In contrast, PlyA2-EGFP could bind to helical structures when the protein was added during their formation. This indicates that PlyA2 does not inhibit helix formation. As it was shown previously, lipid binding proteins can only bind a few % of target lipids due to steric hindrance^31^, it is thus envisaged that only a limited amount of PlyA2 binds to the forming helices, without significantly affecting the overall lipid assembly.

Although GalCer is enriched in myelin, inhibition of GalCer biosynthesis did not inhibit myelin formation^32,33^. Nevertheless, myelin abnormalities such as reduced myelin thickness, redundant myelin outfoldings and vacuole formation were detected in mutant mice^32,33^. It is interesting to note that mice with double deficiency of GalCer synthase and fatty acid 2-hydroxylase form myelin enriched with SM. Currently it is proposed that GalCer is required for the stability and maintenance of myelin during ageing^34^.

CPE is required for the ensheathment of peripheral nerves in *Drosophila*^6,22^. While it cannot be excluded that CPE is involved in ensheathment via direct interaction with regulating proteins, both the multilayer and tight packing nature of CPE:PC membranes suggest that CPE directly supports and stabilizes the glial membranes wrapped around neurons in *Drosophila*. It is interesting that similar to GalCer in mammalian myelin, replacement of CPE with SM rescued cortex glial abnormalities in *Drosophila*^22^. This suggests that similar to GalCer, CPE may be involved in the long term stability of glial membranes or in pathological conditions.

## Materials and Methods

### Materials

*N*-acyl sphingosylphosphorylethanolamine (buttermilk, bovine; ceramide phosphoethanolamine, milk CPE) was obtained from Matreya (Pleasant Gap, PA). *N*-lauroyl sphingosylphosphorylethanolamine (d17:1/*N*-12:0 CPE), palmitoyl sphingomyelin (d18:1/*N*-16:0 SM), 1,2-dioleoyl-*sn*-glycero-3-phosphocholine (diC18:1 PC), 1,2-dipalmitoyl-*sn*-glycero-3-phosphocholine (diC16:0 PC), 1-palmitoyl-2-oleoyl-*sn*-glycero-3-phosphocholine (C16:0, C18:1 PC), 1,2-dioleoyl-*sn*-glycero-3-phosphoethanolamine (diC18:1 PE), 1,2-dipalmitoyl-*sn*-glycero-3-phosphoethanolamine (diC16:0 PE) and 1,2-dioleoyl-*sn*-glycero-3-phosphoserine (diC18:1 PS) were purchased from Avanti Polar Lipids (Alabaster, AL). *N*-oleoyl-_D_-sphingomyelin semisynthetic from bovine brain sphingomyelin (*N*-18:1 SM) was from Sigma. 1,1′-dioctadecyl-3,3,3′,3′-tetramethylindocarbocyanine perchlorate (DiI C18) was from Life Technologies (Carlsbad, CA). 1,6-diphenyl-1,3,5-hexatriene (DPH) was from Koch-Light Laboratories (Colnbrook, UK). *N*-lignoceroyl sphingosylphosphorylethanolamine (d18:1/*N*-24:0 CPE) and *N*-nervonoyl sphingosylphosphorylethanolamine (d18:1/*N*-24:1^Δ15 (c)^ CPE) were custom synthesized by Avanti Polar Lipids. *N*-palmitoyl sphingosylphosphorylethanolamine (d18:1/*N*-16:0 CPE) and *N*-oleoyl sphingosylphosphorylethanolamine (*N*-18:1 CPE) were enzymatically synthesized from d18:1/*N*-16:0 SM and *N*-18:1 SM as described previously^16^ and purified by thin layer chromatography (TLC). Both d18:1/*N*-16:0 CPE and *N*-18:1 CPE gave a single spots on TLC stained with Dittmer or ninhydrin reagents. Lipids were quantitated by phosphate analysis. Pleurotolysin A2-EGFP (PlyA2-EGFP)^7^ and mCherry-non toxic-lysenin (mCherry-NT-Lys)^21,35^ were prepared as described previously.

### Preparation of lipid suspensions

Lipid suspensions were prepared by gentle hydration of lipid films. To this end, CPE stock solutions were prepared in chloroform:methanol (2:1) while all other lipid stock solutions were in chloroform only. The stock solutions were mixed in a glass test tube at the desired molar ratio (total 150 n moles), dried under N_2_ flow and kept *in vacuo* at least for 2 h. Removal of trace organic solvent from lipid film is crucial to obtain helical structures. 500 μL MilliQ water (Millipore, Japan) or PBS (1.058 mM KH_2_PO_4_, 2.96 mM Na_2_PO_4_, 155 mM NaCl, pH 7.2) was added to the lipid film at room temperature (21–22 °C) and the resulting mixture was incubated at the indicated temperatures for 3 h to prepare the lipid suspension. The lipid suspensions were observed by darkfield or fluorescence microscopy at room temperature. Alternatively, the lipid suspensions were further processed for electron microscope observation.

### Gas chromatography

CPE was transmethylated with boronfluoride-methanol reagent 14% at 100 °C for 90 min as previously described^36^. The resulting fatty acid methyl esters (FAME) were extracted with hexane, and analyzed on a Shimadzu GC-14A gas chromatograph using an Omegawax 320 (30 m × 0.32 mm × 0.25 μm) capillary column (Supelco, Bellefonte, PA, USA) and an oven temperature programmed from 100 °C to 250 °C with helium as a carrier gas^36^. FAME peaks were identified by their retention times compared to known standards and the results expressed in mol %.

### Differential scanning calorimetry (DSC)

The lipid films were prepared as outlined above and hydrated with HEPES buffer (20 mM HEPES at pH 7.0, 100 mM NaCl, 100 mM EDTA) at a final concentration of 0.5 mM. After three freeze-thaw cycles (each cycle: −80 °C, 15 min; 60 °C, 15 min; 30 s vortex mixing), the hydrated lipid samples and reference buffer solutions were degassed *in vacuo* for at least 5 min (Microcal ThermoVac vacuum pump) prior to loading into the GE Healthcare MicroCal VP-DSC Microcalorimeter. Samples were scanned at a rate of 10 degrees/h over a range of 20–80 °C with 10 repeat cycles. The data of the 10^th^ heating curve was processed with Origin 7 software.

### Anisotropy measurement

DPH anisotropy measurements were performed on a Fluorolog spectrofluorometer (Horiba, Kyoto, Japan), operating in the T format as reported previously^37^ with slight modifications. The lipids were mixed with DPH at a molar ratio of 1/200 and dried under N_2_ gas. The dry samples were kept in high vacuum for at least 1 h. Lipid suspensions were prepared by hydrating 100 or 200 mmol/L lipids in water at 60 °C. The suspensions were sonicated using a bath sonicator (US-1A, As One, Osaka, Japan) for 5 min. Samples were repeatedly scanned three times within the indicated temperature ranges at a rate of 1 degree/min. Excitation and emission wavelengths were 357 and 451 nm, respectively. The steady-state anisotropy, *r*, was determined from the 4^th^ scan as described^38^.

### Darkfield microscopy and fluorescence microscopy

The shape of lipid assemblies in suspension was observed by darkfield microscopy^39^. The darkfield microscope (Eclipse E600, Nikon, Japan) was equipped with two objectives (100x, N.A. = 0.5–1.3 and 40x, N.A. = 0.75) and a darkfield condenser (N.A. = 1.0–1.4). Images were captured with a sCMOS camera (ORCA-Flash4.0, Hamamatsu photonics, Japan) and processed using imageJ (NIH, USA). Simultaneous observation of darkfield and epifluorescence images was performed with the same objective and appropriate filter units (B3A for EGFP and G2A for DiI C18 and mCherry).

### Electron microscopy

Negative staining electron microscopy was performed as reported previously^30^ with some modifications. Lipid suspensions were adsorbed onto poly-D-lysine-treated pioloform-coated specimen grids and negatively stained with 2% uranyl acetate. The samples were examined by transmission electron microscope (JEM1230, JEOL, Japan) with the help of the Materials Characterization Team in RIKEN Advanced Technology Support Division. Electron micrographs were recorded with a CCD camera (Veleta, Olympus-SIS, Germany).

## References

[1] Yunis EJ, Lee RE (1970). Tubules of globoid leukodystrophy: a right-handed helix. Science.

[2] Archibald DD, Yager P (1992). Microstructural polymorphism in bovine brain galactocerebroside and its two major subfractions. Biochemistry.

[3] Kulkarni VS, Anderson WH, Brown RE (1995). Bilayer nanotubes and helical ribbons formed by hydrated galactosylceramides: acyl chain and headgroup effects. Biophys J.

[4] Kulkarni VS, Boggs JM, Brown RE (1999). Modulation of nanotube formation by structural modifications of sphingolipids. Biophys J.

[5] Freeman MR, Doherty J (2006). Glial cell biology in Drosophila and vertebrates. Trends Neurosci.

[6] Ghosh A (2013). A Global *In Vivo* Drosophila RNAi Screen Identifies a Key Role of Ceramide Phosphoethanolamine for Glial Ensheathment of Axons. PLoS Genetics.

[7] Bhat HB (2015). Evaluation of aegerolysins as novel tools to detect and visualize ceramide phosphoethanolamine, a major sphingolipid in invertebrates. FASEB J.

[8] Hullin-Matsuda F, Makino A, Murate M, Kobayashi T (2016). Probing phosphoethanolamine-containing lipids in membranes with duramycin/cinnamycin and aegerolysin proteins. Biochimie.

[9] Vacaru AM, van den Dikkenberg J, Ternes P, Holthuis JC (2013). Ceramide phosphoethanolamine biosynthesis in Drosophila is mediated by a unique ethanolamine phosphotransferase in the Golgi lumen. J Biol Chem.

[10] Sutterwala SS (2008). Developmentally regulated sphingolipid synthesis in African trypanosomes. Mol Microbiol.

[11] Tafesse FG (2014). Sphingomyelin synthase-related protein SMSr is a suppressor of ceramide-induced mitochondrial apoptosis. J Cell Sci.

[12] Bickert A (2015). Functional characterization of enzymes catalyzing ceramide phosphoethanolamine biosynthesis in mice. J Lipid Res.

[13] Maxfield FR (2002). Plasma membrane microdomains. Curr Opin Cell Biol.

[14] Lingwood D, Simons K (2010). Lipid Rafts As a Membrane-Organizing Principle. Science.

[15] Hullin-Matsuda F, Taguchi T, Greimel P, Kobayashi T (2014). Lipid compartmentalization in the endosome system. Seminar Cell Dev Biol.

[16] Terova B, Heczko R, Slotte JP (2005). On the importance of the phosphocholine methyl groups for sphingomyelin/cholesterol interactions in membranes: a study with ceramide phosphoethanolamine. Biophys J.

[17] Shirota K (2016). Detection of Sphingomyelin Clusters by Raman Spectroscopy. Biophys J.

[18] Ramstedt B, Slotte JP (2006). Sphingolipids and the formation of sterol-enriched ordered membrane domains. Biochim Biophys Acta.

[19] Ishitsuka R, Kobayashi T (2004). Lysenin: A new tool for investigating membrane lipid organization. Anat Sci Int.

[20] Shinitzky M, Barenholz Y (1978). Fluidity parameters of lipid regions determined by fluorescence polarization. Biochim Biophys Acta.

[21] Abe M (2012). A role for sphingomyelin-rich lipid domains in the accumulation of phosphatidylinositol 4,5-bisphosphate to the cleavage furrow during cytokinesis. Mol Cell Biol.

[22] Kunduri G (2018). Defective cortex glia plasma membrane structure underlies light-induced epilepsy in cpes mutants. Proc Natl Acad Sci USA.

[23] Sripada PK, Maulik PR, Hamilton JA, Shipley GG (1987). Partial synthesis and properties of a series of N-acyl sphingomyelins. J Lipid Re.

[24] Van Dijck PW, De Kruijff B, Van Deenen LL, De Gier J, Demel RA (1976). The preference of cholesterol for phosphatidylcholine in mixed phosphatidylcholine-phosphatidylethanolamine bilayers. Biochim Biophys Acta.

[25] Marsh, D. *Handbook of lipid bilayers*. 2nd edition edn (CRC Press, 2013).

[26] Guan XL (2013). Biochemical membrane lipidomics during Drosophila development. Dev Cell.

[27] Curatolo W, Neuringer LJ (1986). The effects of cerebrosides on model membrane shape. J Biol Chem.

[28] John K, Schreiber S, Kubelt J, Herrmann A, Muller P (2002). Transbilayer movement of phospholipids at the main phase transition of lipid membranes: implications for rapid flip-flop in biological membranes. Biophys J.

[29] Yager, P., Chappell, J. & Archibald, D. D. In *Biomembrane Structure and Function-The State of the* Art (eds Gaber, B. P. & Easwaren, K. R. K.) 1–18 (Adenine Press, Schnectady, New York, 1992).

[30] Iwamoto K (2007). Curvature-dependent recognition of ethanolamine phospholipids by duramycin and cinnamycin. Biophys J.

[31] Hullin-Matsuda F, Murate M, Kobayashi T (2018). Protein probes to visualize sphingomyelin and ceramide phosphoethanolamine. Chem Phys Lipids.

[32] Coetzee T (1996). Myelination in the absence of galactocerebroside and sulfatide: normal structure with abnormal function and regional instability. Cell.

[33] Bosio A, Binczek E, Stoffel W (1996). Functional breakdown of the lipid bilayer of the myelin membrane in central and peripheral nervous system by disrupted galactocerebroside synthesis. Proc Natl Acad Sci USA.

[34] Schmitt S, Castelvetri LC, Simons M (2015). Metabolism and functions of lipids in myelin. Biochim Biophys Acta.

[35] Abe M, Kobayashi T (2017). Dynamics of sphingomyelin- and cholesterol-enriched lipid domains during cytokinesis. Methods Cell Biol.

[36] Hullin-Matsuda F (2007). De novo biosynthesis of the late endosome lipid, bis(monoacylglycero)phosphate. J Lipid Res.

[37] Halling KK, Ramstedt B, Nystrom JH, Slotte JP, Nyholm TK (2008). Cholesterol interactions with fluid-phase phospholipids: effect on the lateral organization of the bilayer. Biophy J.

[38] Lakowicz, J. R. *Principles of Fluorescence Spectroscopy, 2nd Ed*. (Kluwer Academic/Plenum Publishers, 1999).

[39] Inaba T (2016). Phospholipase Cbeta1 induces membrane tubulation and is involved in caveolae formation. Proc Natl Acad Sci USA.

